# The correlation of honesty-humility and learning goals with academic cheating

**DOI:** 10.1007/s11218-022-09742-2

**Published:** 2022-12-05

**Authors:** Nina Reinhardt, Lina-Marie Trnka, Marc-André Reinhard

**Affiliations:** Department of Psychology, University of Kassel, Holländische Str. 36–38, 34127 Kassel, Germany

**Keywords:** Academic cheating, Honesty-Humility, Learning goals, Achievement goal theory

## Abstract

Academic cheating is a problem that affects many educational institutions and has become increasingly significant with the new challenges of online education. Recent studies have found that learning goals are correlated with cheating behavior among students. In this study, we investigated whether learning goals are still a predictor of cheating behavior when controlling for students’ Honesty-Humility (emanated from the HEXACO model of personality) within a sample of 311 German university students. Regrading students’ learning goals, we assessed their learning approach, performance approach, performance avoidance, and work avoidance. The result shows an intermediate negative and highly significant association between Honesty-Humility and academic cheating. Learning goals did not explain any incremental variance in academic cheating that goes beyond the Honesty-Humility factor. As the only exception, the work avoidance goal was found to also predict cheating behavior, but this positive association seems to be not as strong as the negative correlation between Honesty-Humility and academic cheating. We discuss the theoretical and practical implications of these results and make recommendations for future research.

## Introduction

Academic cheating has been a popular field of research in many psychological disciplines, such as educational and social psychology. Following Newstead et al. (1996), this study defines academic cheating as cheating on coursework including plagiarism, data manipulation and collaborative cheating, cheating on exams including collusion, lying for special consideration (for example lying for extension), and noncollaborative cheating in exams (for example writing off something). Academic cheating can cause severe consequences; for example, cheating negatively affects a student’s ethical and moral standards. However, non-cheaters are also affected because they may feel unfairly treated when they are graded worse compared to academic cheaters (Iqbal et al., 2021). Thus, institutions must react to the misconduct. Cheating can also impact the education system as a whole, undermining the validity of academic tests (Daumiller & Janke, 2019; Garavalia et al., 2007; McCabe, 2005; McCabe et al., 2001). Research has shown that cheating during an academic career influences and predicts cheating and counterproductive behavior in a future workplace (Nonis & Swift, 2001). Therefore, it is important to investigate academic cheating and possible predictors, as cheating has broad implications and consequences.

In light of the ongoing COVID-19 pandemic, the importance of investigating academic cheating has increased, and past research has revealed that compared to in-person classes, academic cheating is more likely in online classes (Kennedy et al., 2000). Similarly, King and Case (2014) have reported a trend toward increased cheating in online environments. Watson and Sottile (2008), however, have reported higher actual cheating rates in live classes, even though students self-reported they were more likely to cheat in online classes. Grijalva et al. (2006) have identified no difference between the cheating rates in online versus live classes. Overall, these findings are inconsistent but may simply indicate the need for more research in this field.

Because cheating is often described as motivated behavior as it involves a conscious decision to break rules to gain an advantage (Anderman, 2007; Anderman & Koenka, 2017; Anderman & Murdock, 2007; Daumiller & Janke, 2020; McCabe et al., 2001; Schraw et al., 2007), students’ motivation is assumed to play a role in whether they decide to cheat.

### Learning goal theory

One motivational approach to explain academic cheating is the learning goal theory, also labeled the achievement goal theory. Learning goals describe what motivates students to put effort into their work, and these different aims are assumed to lead to differential performance outcomes (e.g., Elliot et al., 2005). In past research, a dichotomous distinction between a *mastery (or learning) goal orientation* and a *performance (or extrinsic) goal orientation* was prominent (Dweck, 1986; Dweck & Leggett, 1988; Elliot, 2005). The mastery goal orientation is described as an adaptive pattern by students who want to learn the contents and gain a deep understanding of them (Dweck, 1986; Elliot, 2005). By contrast, the performance goal orientation is described as a maladaptive, helpless pattern, with a focus on demonstrating competence compared with others as a means to aggrandize one’s ability status (Daumiller & Janke, 2020; Dweck, 1986; Elliot, 2005). As research developed, further (more detailed) distinctions have been discussed.

As an example, Elliot (2005) applied a trichotomous approach. Besides the mastery goal, defined as the student’s motivation of being geared toward expansion of personal knowledge, a deeper understanding of the studied subjects, and personal improvement, Elliot further separated the performance goal into a *performance approach goal* and a *performance avoidance goal*. Students with a strong performance approach goal orientation strive to achieve a specific result, such as recognition for their performance or grades or a positive comparison to others. The aim is to appear competent and to achieve a positive outcome or accomplishment. Students with a strong performance avoidance goal orientation are mainly anxious about not being seen as competent and try to avoid negative comparisons. The aim here is to not be seen as incompetent and therefore to avoid a negative outcome. In 2001, Elliot and McGregor proposed a 2 (mastery vs. performance) x 2 (approach vs. avoidance) goal framework, which further separates the mastery goal into a mastery approach goal and a mastery avoidance goal; the main difference is the valence of competence, where competence in mastery approach goals is valenced positive, and competence in mastery avoidance goals is valenced negative.

Key to the present work is previous research that has shown how a student’s goal orientation predicts academic cheating. For example, students with a performance goal orientation are more likely to cheat in the academic context, compared with students with a mastery approach goal orientation, independent of the approach or the avoidance orientation (Jordan, 2001; Van Yperen et al., 2011; see also Rettinger et al., 2004). The basic argument is that cheating for students who are mastery-oriented would not assist them in their goal to truly understand the learned content. By contrast, for students, who are performance-oriented, independent of approach or avoidance oriented, cheating would help to achieve their goal (i.e., approach success to others, respectively avoid failure; e.g., Anderman, 2007).

More recent studies have shown that many other variables need to be considered when investigating the effect of learning goals on cheating behavior. For example, Daumiller and Janke (2020) have highlighted the importance of social norms for this effect, revealing that if the environment shows acceptance for the cheating behavior, cheating increases. These authors have also shown that the focus of evaluating performance interacts with the learning goal orientation (Daumiller & Janke, 2019); when students’ results rather than their learning processes were the focus of performance evaluation, cheating behavior increased. Anderman and Won (2017) have asserted that the perceived goal structure of the classroom (i.e., mastery being emphasized in the classroom by the teachers, rather than just grades and performance) also affects the students’ beliefs about cheating.

Based on the learning goal theory, academic cheating seems to be a highly motivated behavior, but there are also other characteristics (independent of students’ learning goals) that affect dishonest behavior. Besides characteristics such as age, gender, or cultural differences, which have already been investigated (McCabe et al., 2001; Miller et al., 2007), there are solid theoretical arguments for the personality factor Honesty-Humility (emanated from the HEXACO model of personality; Lee & Ashton 2004) to also predict academic cheating.

### Honesty-Humility

In the past, the most popular approach to measure a person’s personality consisted of the five-factorial personality model (i.e., the Big Five), which differentiates between the personality traits Neuroticism, Extraversion, Openness to experiences, Consciousness, and Agreeableness. Among these personality factors, Agreeableness and Consciousness are the best predictors for academic performance and also for academic cheating (Cuadrado et al., 2021; De Vries et al., 2011). Importantly, recent research revealed a six-factorial personality model, labeled as the HEXACO model of personality (Lee & Ashton, 2004), that comprises a sixth personality factor denoted as Honesty-Humility. Honesty-Humility is described as “the tendency to be fair and genuine in dealing with others, in the sense of cooperating with others even when one might exploit them without suffering retaliation” (Ashton & Lee, 2007, p. 156). This factor includes the four facets Sincerity (i.e., the tendency to be genuine in interpersonal relations), Fairness (i.e., the tendency to avoid fraud and corruption), Greed avoidance (i.e., the tendency to be uninterested in possessing lavish wealth, luxury goods, and signs of high status), and Modesty (i.e., the tendency to be modest and unassuming).

In the past, Honesty-Humility appeared to be the key factor in predicting dishonesty, with people lower in Honesty-Humility showing increased dishonest behavior (Heck et al., 2018; Hilbig & Zettler, 2015; Schild et al., 2020). Honesty-Humility has also been found to accurately predict academic cheating. De Vries et al. (2011) also showed Honesty-Humility to be the key predictor of counterproductive academic behavior, including cheating and plagiarism. The work of Hilbig and Zettler (2015) revealed Honesty-Humility as a predictor of cheating whose impact goes beyond any other factors in the HEXACO model or the Five Factor model. Pfattheicher et al. (2019) also demonstrated the predictive validity of Honesty-Humility for cheating behavior and further showed that Honesty-Humility overshadowed other relevant variables for predicting dishonesty (i.e., narcissism, Machiavellianism, psychopathy, and sadism). O’Connor et al. (2021) examined cheating behavior across different adult age groups, and their results showed that higher Honesty-Humility predicted less cheating behavior. In line with these studies, a re-analysis of several studies on cheating found Honesty-Humility to be a reliable and robust predictor for cheating behavior in general (Heck et al., 2018). Among the four facets of the Honesty-Humility factor, Fairness was the most accurate predictor for academic cheating, whereas Greed avoidance best predicted the specific cheating behavior of collegiate cheating (De Vries et al., 2011; Van Rensburg et al., 2018).

### The present study

Following past research, learning goals are a valid predictor for academic cheating (Janke et al., 2019; Jordan, 2001; Rettinger et al., 2004; Van Yperen et al., 2011). Interestingly, Dinger et al. (2015) have demonstrated a correlation between Honesty-Humility and learning goals. Under the assumption that people higher in Honesty-Humility do not feel entitled to more respect than others, as hypothesized, their results showed significant negative correlations between Honesty-Humility and both the performance approach goal and the performance avoidance goal, and a significant positive correlation between Honesty-Humility and the mastery goal. But in sum, only a few researchers have pursued the direct influence of Honesty-Humility on learning goals.

Given the recent findings revealing Honesty-Humility to be a key factor in predicting general dishonest behavior (Heck et al., 2018; Hilbig & Zettler, 2015; Schild et al., 2020), but particularly with reference to research that revealed (a) Honesty-Humility to significantly predict (academic) cheating (Ashton & Lee, 2008; De Vries et al., 2011; Heck et al., 2018; Hilbig & Zettler, 2015; O’Connor et al., 2021; Pfattheicher et al., 2019) and (b) Honesty-Humility to share a substantial amount of variance with learning goals (Dinger et al., 2015), we wanted to test if learning goals could explain any significant incremental variance of academic cheating that goes beyond the explained variance of the predictor Honesty-Humility.

In this study, we used two different self-report scales to measure academic cheating (i.e., Anderman & Won, 2017; Rettinger et al., 2004). Both self-report scales trace back to Newstead et al. (1996), who operationalized academic cheating as cheating on coursework including plagiarism, data manipulation and collaborative cheating, cheating on exams including collusion, lying for special consideration (for example lying for extension), and noncollaborative cheating in exams (for example writing off something).

For measuring student’s learning goals, the present work employs Elliot’s (2005) approach of a trichotomous goal structure. This approach includes the mastery (or learning) goal, the performance approach goal, and the performance avoidance goal. We refrain from a further separation of the mastery goal into a mastery approach goal and a mastery avoidance goal as suggested by Elliot and McGregor (2001), because we believe the global mastery goal should negatively predict academic cheating—whether approach or avoidance-oriented—as this global mastery goal should encourage students to truly improve their learning in any case and therefore lead to decreased academic cheating (e.g., Anderman, 2007; Janke et al., 2019; Van Yperen et al., 2011). Regarding the separation of the performance goal into a performance approach goal and a performance avoidance goal, some researchers would likely argue similarly, claiming that a global measurement of the construct is sufficient for the prediction of academic cheating (e.g., Van Yperen et al., 2011). However, in the context of academic cheating, there is first evidence revealing that both performance goals (i.e., the performance approach goal and the performance avoidance goal) differ in the way that the performance avoidance goal is more closely related to academic cheating compared to the performance approach goal. Indeed, the results of Janke et al. (2019) showed that academic cheating (or rather the use of questionable research practices which are defined as strategies that aim to increase the chance to publish at the cost of scientific accuracy) is positively linked to the performance approach goal but negatively linked to the performance avoidance goal (Janke et al., 2019). To test this, on the one side, positive association, and on the other side negative association between both performance goals and academic cheating, we followed the trichotomous goal structure of Elliot (2005).

Additionally, we included the so-called *work avoidance goal orientation* in our research, which is defined as the motivation to achieve good results with little effort and workload (Daumiller et al., 2019; Elliot, 2005). We included this learning goal in addition to the trichotomous structure because previous research has shown avoidance goal orientation to be highly relevant in predicting students’ academic behavior in various ways. King and McInerney (2014) showed that the work avoidance goal can be associated with several negative outcomes in the academic context, such as lower grades and less engagement. Furthermore, they demonstrated a clear distinction of the work avoidance goal from the mastery as well as the performance goals. Pavlin-Bernardić et al. (2017) examined cheating behavior among students and found a significant positive association between the work avoidance goal and active cheating to increase the own academic outcome.

#### Hypotheses

In more detail, we expect Honesty-Humility to be a significant predictor of academic cheating (*Hypothesis 1*). Even if this is not preregistered, a clear direction of the relationship can be predicted: Students lower in Honesty-Humility should report increased academic cheating. We also predict learning goals to be a significant predictor for academic cheating (*Hypothesis 2*). Again, even not preregistered, based on previous research we hypothesize that students lower in their mastery goal orientation should report increased academic cheating. Even if recent findings are mixed (cf., Janke et al., 2019), we further predict that students higher in their performance approach orientation, and higher in their performance avoidance orientation, should report increased academic cheating. We also predict a positive correlation between work avoidance orientation and academic cheating. Moreover, following the argument of Janke et al., (2019), one could predict that all learning goals could explain significant incremental variance of academic cheating that goes beyond the explained variance of Honesty-Humility. By contrast, following the theoretical reasoning of Hilbig and Zettler (2015; see also, Pfattheicher et al., 2019), we do not expect (and preregistered) that learning goals do explain any significant incremental variance of academic cheating that goes beyond the explained variance of the predictor Honesty-Humility (*Hypothesis 3*).

## Method

Before data collection, the study was preregistered at *AsPredicted* (https://aspredicted.org/yb2k9.pdf). The *Open Science Framework* (OSF; osf.io/tcen4) entails data, syntax, and Supplemental Material including detailed information about further preregistered analyses. For the study, relevant ethical guidelines were followed.

### Subjects

Before data collection, we conducted an a priori power analysis to determine the minimum sample size required to detect the expected effect. We used the program G*Power (Version 3.1.9.4; Faul et al., 2009). With an assumed power of 0.80, setting Type I error rate at *p* < .05, and assuming an effect size between learning goals and academic cheating (*Hypothesis 2*) of *r* = .16 (cf. Janke et al., 2019), the power analysis for correlation (two-tailed) revealed a minimal sample size of *N* = 237. Nevertheless, we aim to collect data from *N* = 250 participants. To check whether this sample size is also adequate for detecting a potential correlation between Honesty-Humility and academic cheating (*Hypothesis 1*), we additionally conducted a posthoc power analysis. With the given sample size of *N* = 250, and assuming an effect size between Honesty-Humility and cheating of *r* = .30 (Heck et al., 2018), the posthoc power analysis for correlation (Type I error rate at *p* < .05, two-tailed) revealed a power > 0.99.

Data collection began in November 2021. We set a data collection period of six weeks, wherein we actively recruited participants. In the preregistration, we stated that if we did not achieve the minimum sample size after the set period, we would continue with the data collection for an unknown period until we had collected the data of at least 250 participants. However, after six weeks, sufficient participants were recruited. Recruiting took place via *Surveycircle*, which is an online platform with a nonmonetary function that recruits participants to take part in research projects (https://www.surveycircle.com/de/). We compensated participants who took part in the survey via this website with so-called Surveycircle points. Psychology students from the University of Kassel were compensated with points for participation required for their course credits. The final sample consisted of *N* = 311 German university students (76.8% female, 22.2% male, 1% diverse) with a mean age of 24.4 years (*SD* = 6.25).

### Procedure

Participants first read the informed consent, including the prerequisites to participate in the study (i.e., over 18 years, registered student at a German university at the date of participation) and a declaration of voluntariness. Participants were informed that their responses would remain anonymous. After the participants agreed to the informed consent, they completed two different scales for the measurement of academic cheating, followed by the Honesty-Humility scale. Next, participants completed the four subscales to measure students’ mastery (or learning) goal orientation, performance approach goal orientation, avoidance approach goal orientation, and work avoidance goal orientation. Finally, participants answered demographic questions.

### Measures

#### Academic cheating

First, we used 17 self-adapted items (α = 0.73) of a scale created by Rettinger et al. (2004). This instrument measures cheating behaviors on exams, papers, and homework/labs. It is mainly about using unauthorized information in different test situations, respectively giving this information to others. For example, “I copied from someone during an in-class exam”, “I gave test information to someone in a later section”, and “I used exact words or ideas from a book or other printed publication without acknowledging the source”.

Second, we used 22 self-adapted items (α = 0.71) of a scale created by Anderman and Won (2017). Next to common cheating behaviors like plagiarism and the use (and distribution) of unauthorized information in test situations, this scale additionally assesses further aspects like, for example, making false personal excuses (“Lying about medical or other circumstances to get an extended deadline or exemption from a piece of work”) and collaborative cheating (“In a situation where students mark each other’s work, coming to an agreement with another student or students to mark each other’s work more generously than it merits”).

Both scales required participants to indicate if they had ever engaged in the described behavior (yes or no). The time period was not further defined, so that the students could align their answer to the entire university career—and possibly also to a previous school career. For both scales, all “yes” answers were computed to a cheating score ranging from zero to 17 for the scale created by Rettinger et al., and from zero to 22 for the scale created by Anderman and Won. In contrast to the preregistration protocol but following one anonymous suggestion of one reviewer, we summarized both cheating scores to one final dependent variable; this variable ranges from zero to 39 and was labeled as *cheating*. Internal reliability across both scales was α = 0.84.

#### Learning goals

To measure students’ learning goal orientation, we used 16 self-adapted items of Instructors’ Achievement Goals for Teaching scale created by Daumiller et al. (2019). As our target group consisted of German students, we adapted the items by changing certain words to fit for students instead of teaching trainees. Mastery goal orientation was measured with four items (α = 0.93; “I want to constantly improve my competences”); Daumiller denoted it as a *learning approach*. Performance approach was measured with the four items (α = 0.94) of Daumillers’ subscale denoted as *task approach* (“I want to fulfill the different requirements very well”). Performance avoidance was measured with the four items (α = 0.88) of Daumillers’ subscale denoted as *task avoidance* (e.g., “I want to avoid being bad”), and *work avoidance* was also measured with four items (α = 0.95; e.g., “It is important to me to have little to do”). Participants were instructed to indicate their agreement on each statement on an 8-point scale ranging from 1 (*do not agree*) to 8 (*agree completely*).

#### Honesty-Humility

We measured Honesty-Humility with the 16 relevant items (α = 0.82) of the HEXACO-PI-R (100-item version) created by Lee and Ashton (2018). Participants were instructed to indicate their agreement on each statement on a 5-point scale ranging from 1 (*strongly disagree*) to 5 (strongly *agree*).

## Results

As shown in Table 1, and as predicted in *Hypothesis 1*, Honesty-Humility was significantly negatively correlated with academic cheating (*r*_*p*_ = − 0.31, 95% CI = [-0.40; -0.20], *p* < .001). Regarding the different learning goals, task avoidance was significantly negatively correlated with cheating (*r*_*s*_ = − *.1*2, 95% CI = [-0.23; -0.01], *p* = .035), and work avoidance was significantly positively correlated with cheating (*r*_*p*_ = 0.18, 95% CI = [0.07; 0.29], *p* = .001). No other significant correlations between the different learning goals and cheating appeared (all *ps ≥* 0.057). Thus, *Hypothesis 2* was only supported regarding students’ work avoidance goal. Even there was a significant correlation between task avoidance and cheating, this was against our predicted direction, indicating students stronger in their task avoidance orientation to report decreased cheating. As preregistered, we also conducted a correlation analysis with both cheating scales treated separately (see the Supplemental Material).

**Table 1:** *Means, Standard Deviations, Intercorrelations and Confidence Intervals for Study Variables*

Variables	Mean	*SD*	range		(1)	(2)	(3)	(4)	(5)	(6)
(1) Cheating	6.84	4.95	1–39		−					
(2) Learning approach	6.83	1.17	1–8		-.08^a^ [-0.19; 0.04]	−				
(3) Task approach	6.92	1.11	1–8		-.11^a^ [-0.22; 0.01]	0.52***^a^ [0.43; 0.60]	−			
(4) Task avoidance	7.04	1.19	1–8		-0.12*^a^ [-0.23; -0.01]	0.29***^a^ [0.18; 0.39]	0.57***^a^ [0.49; 0.64	−		
(5) Work avoidance	4.34	1.80	1–8		0.18** [0.07; 0.29]	-0.28***^a^ [-0.38; -0.17]	-0.23***^a^ [-0.33; -0.12	-.06^a^ [-0.18; 0.05]	−	
(6) Honesty-Humility	3.52	0.58	1–5		-0.31*** [-0.40; -0.20]	0.18**^a^ [0.06; 0.29]	0.15**^a^ [0.03; 0.26	0.15*^a^ [0.03; 0.26]	-0.22*** [-0.33; -0.11]	−

*Note. N* = 311. Values in brackets are 95% confidence intervals. Cheating = Summarized score of both cheating scales.

^a ^Because of the extreme left-skewed distribution of learning approach (skewness = -1.02, *SE* = 0.14), task approach (skewness = -1.23, *SE* = 0.14), and task avoidance (skewness = -1.41, *SE* = 0.14), we calculated Spearman’s rank-order correlations for correlation coefficients involving these variables.

*p < .05, two-tailed. **p < .01, two-tailed. ***p < .001, two-tailed.

To perform the regression analyses, we implemented the bootstrapping method, which is a nonparametrical procedure and robust against violations in the distributional assumptions. We performed this procedure by generating 2,000 bootstrap samples and by using the BCa method (Field, 2013).

We conducted linear regression models using Honesty-Humility as predictor for the summarized score of both cheating scales (Model 1). In a second step, we inserted the variables learning approach, task approach, task avoidance, and work avoidance as predictors (Model 2) to determine if these additional predictors explain incremental variance.

As shown in Table 2 and in line with *Hypothesis 1*, Honesty-Humility significantly predicted academic cheating in Model 1, *B* = -2.60, *SE B* = 0.48, BCa 95% CI = [-1.79; -0.65], β = -0.31, *p* < .001. Supporting our *Hypothesis 1*, this negative association remained robust in Modul 2, *B* = -2.32, *SE B* = 0.47, BCa 95% CI = [-3.26; -1.43], β = -0.27, *p* < .001. This analysis revealed weak support for *Hypothesis 2*; in Model 2, only one of the inserted learning goals was found to be a significant predictor. Only work avoidance showed a small significant effect on academic cheating, *B* = 0.33, *SE B* = 0.16, BCa 95% CI = [0.03; 0.66], β = 0.12, *p* = .037. In line with *Hypothesis 3*, including the learning goals learning approach, task approach, and task avoidance in our Model 2 did not explain any incremental variance beyond the Honesty-Humility factor (see Table 2).

**Table 2:** *Regression Coefficients on Academic Cheating*

Model	Predictor							
				BCa 95% CI				
		*B*	*SE B*	Low	High	β	*R* ^*2*^	Δ*R*^*2*^
(1)	Honesty-Humility	-2.60***	0.48	-3.56	-1.73	-0.31	0.09	0.09***
(2)	Honesty-Humility	-2.32***	0.47	-3.26	-1.43	-0.27	0.11	0.02
	Learning approach	0.17	0.30	-0.45	0.79	0.04		
	Task approach	-0.23	0.33	-0.85	0.37	-0.05		
	Task avoidance	-0.12	0.27	-0.64	0.39	-0.03		
	Work avoidance	0.33*	0.16	0.03	0.65	0.12		

*Note. N =* 311. Results are computed by using the bootstrapping method with 2,000 bootstrap samples and BCa confidence intervals. Cheating = Summarized score of both cheating scales.

*p < .05, two-tailed. **p < .01, two-tailed. ***p < .001, two-tailed.

As preregistered, we also conducted parallel regression analyses (Model 1 and Model 2) but treated both cheating scales separately. For both, the scale of Rettinger et al. (2004; i.e., Scale 1) and the scale of Anderman and Won (2017; i.e., Scale 2), Honesty-Humility was a strong and significant predictor in Model 1 (Scale 1: *B* = -1.22, *SE B* = 0.28, BCa 95% CI = [-1.79; -0.65], β = -0.26, *p* < .001; Scale 2: *B* = -1.39, *SE B* = 0.25, BCa 95% CI = [-1.88; -0.94], β = -0.31, *p* < .001). For both cheating scales, this strong negative association remained robust even when controlling for learning goals in Model 2 (Scale 1: *B* = -1.09, *SE B* = 0.28, BCa 95% CI = [-1.66; -0.52], *p* < .001; *B* = -1.23, *SE B* = 0.24, BCa 95% CI = [-1.71; -0.79], β = -0.28, *p* < .001). For both cheating scales, none of the additional inserted learning goals proved to be a significant predictor with only one exception. When analyzing the cheating scale of Anderman and Won (2017), the work avoidance goal showed a small significant effect on academic cheating, *B* = 0.17, *SE B* = 0.08, BCa 95% CI = [0.01; 0.33], β = 0.12, *p* = .043. A more detailed report of the preregistered regression analyses with both scales individually can be found in the Supplemental Material.

## Discussion

The present study examined the predictive value of Honesty-Humility and learning goals on self-reported cheating behavior of university students. In line with our assumption, we found Honesty-Humility to significantly predict cheating behavior; students lower in Honesty-Humility reported increased academic cheating. The association between Honesty-Humility and academic cheating can be interpreted as an intermediate-sized effect, and it was highly significant. The predictive value of Honesty-Humility remained significant, even when controlling this association for the learning goals learning approach, task approach, task avoidance and work avoidance. Thus, Honesty-Humility appeared to be an important and reliable predictor of academic cheating behavior. This finding is in line with recent research. O’Connor et al. (2021) have also found that as Honesty-Humility scores increase, cheating behavior decreases. Similar conclusions about the importance of Honesty-Humility and its facets in academic dishonesty were drawn by De Vries et al. (2011) and by Van Rensburg et al, (2018).

Regarding the different learning goals, only the learning goal of work avoidance revealed a predicted value that goes beyond the Honesty-Humility factor. We included this specific goal because previous research has shown an association between work avoidance and academic cheating (Pavlin-Bernardić et al., 2017). University students often explain their cheating behavior, even though they know it is wrong, with time pressure and a high workload (Anderman et al., 1998; McCabe et al., 2001; Newstead et al., 1996). Therefore, it seems reasonable to assume that cheating behavior might occur to cut the workload and avoid additional work. However, the found relationship should not be overinterpreted because the found effect can only be interpreted as small; additionally, the confidence interval for this positive association was close to zero. This is further supported by our regression analyses in which we analyzed (as preregistered) the predictive value of Honesty-Humility and learning goals on both cheating scales separately. Here, the positive association between work avoidance and cheating under control of Honesty-Humility was only found regarding one of the two scales. Moreover, and contrary to our prediction, the correlation analysis revealed a significant *positive* association between task avoidance and academic cheating; however, this is in line with recent findings of Janke et al., (2019). Importantly, in none of the conducted regression analyses, task avoidance proved to be a significant predictor when controlling the association for Honesty-Humility. These results strengthen our assumption that the learning goal orientation does not contribute to explaining why some students cheat and others do not, but that Honesty-Humility is the important predictor for these differences.

An alternative approach that may explain the lack of incremental validity of learning goals could be a possible interaction between Honesty-Humility and the learning goal orientation. In one study, Daumiller and Janke (2020) have demonstrated that neither the investigated learning goal nor perceived social norms alone predict cheating, but that the interaction between both variables has a significant effect. The same was demonstrated for the performance goals and performance evaluation (Daumiller & Janke, 2019). In alignment with these findings, Jordan (2001) has found an interaction between motivational variables and different school subjects that predicted cheating. This indicates that an interaction between Honesty-Humility and the learning goal orientation might be considered as an explanation of the mixed and sometimes divergent findings. However, since testing for interactions requires higher sample sizes (Blake & Gangestad, 2020), we refrained from testing a potential interaction between learning goals and Honesty-Humility in our study, but definitely view this as an interesting approach for future research. Anderman and Murdock (2007) have demonstrated that several motivational variables influence the decision to cheat or not to cheat. The authors stated that next to the learning goal orientation, the students’ beliefs and expectations about their own abilities are important, along with the perceived risk of getting caught. Thus, many more variables need to be included to find a model that can fully explain academic cheating.

### Limitations

The present study is the first attempt to examine Honesty-Humility and learning goals on its incremental value for academic cheating. This study poses several limitations that need to be considered when interpreting our results. First, we assumed a power of 80% when determining our minimum sample size based on the association between learning goals and academic cheating. This was only a rough guide, as ours was the first study to examine the listed variables in this constellation, and we had no opportunity to rely on previous research for correct estimates for the power analysis that fit with our study design.

Another limitation of this study is that we used self-reporting measures for all constructs. Different methods to assess cheating behavior might yield more accurate results, as Steger et al. (2020) have proposed. Cheating is an unacceptable behavior, which is often followed by a penalty if disclosed. We can assume that honesty about students’ own cheating behavior is a challenge for most people. Even if the research is carried out for scientific reasons and despite the declaration of anonymity, many students were probably afraid of the consequences of being honest or perhaps also ashamed about their current or past cheating behavior.

Additionally, in the light of the still ongoing Corona pandemic in which online classes are the prevailing method of instruction, certain areas of online cheating were perhaps not directly considered due to selected cheating scales that are designed to measure academic cheating in in-person classes. However, even if some of those “new” online cheating behaviors during online exams (like, for example, searching the internet during a final exam or working on an online exam with several people in the same room without permission) are not explicitly asked, they are covered by the used items.

Finally, we want to mention that although our sample consisted exclusively of (German) students, it is not representative of the field of studies. For example, in our sample, more than half of the participants classified themselves as law, economic, and/or social scientists; this proportion is about five times higher than the proportion among all German students in general. Further, with 76.8% of participants who indicated themselves as female, this proportion is also higher than among German students in general, who have a relatively balanced gender ratio (Statistisches Bundesamt, 2022). In this vein, it could also be beneficial to test different populations against each other (with appropriate academic cheating scales), such as different age groups or students from different study programs. Despite these limitations, the present study poses a good first for future research.

### Implications and future research

The present study contributes to the current state of research by revealing that the association between learning goals and academic cheating, which has been well established in previous research in this field, did not withstand testing against the effect of Honesty-Humility. Honesty-Humility might influence social norms, the attitude toward cheating, or the students’ estimation of their own abilities. It may be useful for future studies in this field to additionally check for interactions and/or to control more established effects for students’ Honesty-Humility.

To increase the generalizability of our findings, future research should rely on different methods when examining academic cheating. Different assessment methods might reveal different results and help expand our knowledge of academic cheating. Furthermore, students’ learning goal orientation can be manipulated via a goal induction (Daumiller & Janke, 2019, 2020) or can be measured via vignette methods (Rettinger & Kramer, 2009; Rettinger et al., 2004). Further,longitudinal studies would be needed to explain the causality behind the examined relationships.

Our study has shown a small effect of the work avoidance goal on academic cheating, but none of the learning goals from the trichotomous goal structure yielded effects. Even if we first basically advised replication of the positive association between work avoidance and academic cheating before giving it too much meaning, this first finding may indicate the importance of further investigation of the learning goals that are less represented in previous research. Even though this study shows that the personal learning goals do not contribute to the explanation of academic cheating beyond the effect of Honesty-Humility, learning goals are important for other topics in the educational context. This study has not addressed the classroom or institutional goal structure. These different levels of goal orientation might influence cheating in other ways than the personal learning goals.

The practical implications of this study concern possible interventions against academic cheating and the identification of cheaters. Previous research has demonstrated there are various ways for educators and institutions to prevent cheating (Anderman et al., 1998; Anderman & Koenka, 2017; McCabe et al., 2001; Stephens, 2008). By identifying which variables have the strongest effects on academic cheating, prevention strategies can be implemented more accurately and can help preserve academic integrity. These adaptions in intervention become even more important in academic cheating in online classes and the new challenges that result from increasing online education. Online education has become increasingly popular and has also become part of daily life for most students due to the COVID-19 pandemic. Because of these developments, research on e-cheating has become urgent. The results of this study and our suggestions for future research could be useful for this research.
